# Correction: A quantitative analysis of the impact of explicit incorporation of recency, seasonality and model specialization into fine-grained tourism demand prediction models

**DOI:** 10.1371/journal.pone.0292825

**Published:** 2023-10-09

**Authors:** The PLOS ONE Staff

There are errors in the Funding section. The correct Funding statement is: This research is partially funded by Conselho Nacional de Desenvolvimento Científico e Tecnológico (CNPQ) and Fundação de Amparo à Pesquisa do Estado de Minas Gerais (FAPEMIG). The authors and grant numbers are as following: Amir Khatibi: (CNPq: 169823/2017-2); Ana Paula Couto da Silva and Marcos A. Gonçalves: (FAPEMIG: PPM-00543-17, PPM-00177-18 and APQ-00262-22), (CNPq: 422593/2018-4, 310538/2020-3, 310668/2020-4, 402711/2021-1 and 403184/2021-5); Jussara Almeida: (CNPq: 305683/2019-5 and 403106/2021-4). The funders had no role in study design, data collection and analysis, decision to publish, or preparation of the manuscript.

The publisher apologizes for the error.
