# Supplementary material for: A quantitative analysis of the impact of explicit incorporation of recency, seasonality and model specialization into fine-grained tourism demand prediction models
Source: PLoS One. 2022 Dec 8;17(12):e0278112. doi: 10.1371/journal.pone.0278112 (PMC9731488; doi:10.1371/journal.pone.0278112)
Supplement: Attachment — Submitted filename: Response_Letter_2nd_revision_PloSOne.pdf [file pone.0278112.s002.pdf]

Paper title: A Quantitative Analysis of the Impact of Explicit Incorporation of Recency, Seasonality and Model Specialization into Fine-Grained Tourism Demand Prediction Models

Autores: Amir Khatibi, Ana Paula Couto da Silva, Jussara Almeida, Marcos A. Gonçalves

Dear Plos One Editor in Chief and Reviewers

We are sending a **second revision version** of our manuscript under submission to **PLOS ONE** Journal as well as the responses to the reviewers' comments.

We would like to sincerely thank all reviewers for their thoughtful and detailed comments that helped improve our paper. We did our best to address all their comments. The original reviewers' comments are in bold, each followed by our responses in italic.

We also performed all the requested editorial changes in our revised manuscript.

Best regards,

Amir Khatibi,
Ana Paula Couto da Silva, Jussara Almeida, Marcos A. Gonçalves

REVIEWER #1

Some final cosmetic comments:

COMMENT #1:

The results of your comparative study should be discussed in-depth and with more insightful comments on the behavior of your algorithm on various case studies. Discussing results should not mean reading out the tables and figures once again.

RESPONSE*: We thank the reviewer for the feedback on our paper.*

As requested, we expanded the Discussion Section with more insightful comments on our experimental results. We also avoided re-reading previous tables and figures and focused on providing more qualitative comments based on our case studies, as suggested.

COMMENT #2:

Avoid lumping references as in [x, y] and all others. Instead summarize the main contribution of each referenced paper in a separate sentence. For scientific and research papers, it is not necessary to give several references that say exactly the same. Anyway, that would be strange, since then what is the innovative scientific contribution of referenced papers? For each thesis state only one reference.

RESPONSE*: We checked for all lumping references in the Related Work Section and broke them to explain the contribution of each separately.*

COMMENT #3:

Avoid using first person.

RESPONSE*: We reduced considerably the use of the first person in the revised manuscript, although we did not remove all uses, since in some points of the text it would sound unnatural or artificial.*

COMMENT #4:

Avoid using abbreviations and acronyms in title, abstract, headings and highlights.

RESPONSE*: We resolved the cases using acronyms in sub-sections in Section 2.4.*

COMMENT #5:

Avoid having heading after heading with nothing in between, either merge your headings or provide a small paragraph in between.

RESPONSE*: We reviewed the paper to guarantee avoiding the issues emphasized by the reviewer. In addition, in the Appendices Section, we made some modifications to avoid heading*

after dividing the Appendices Section into part A and B with a small paragraph explaining each of these sub-sections.

COMMENT #6:

The first time you use an acronym in the text, please write the full name and the acronym in parenthesis. Do not use acronyms in the title, abstract, chapter headings and highlights.

RESPONSE*: We revised the paper and took care of three incidents of this type. Now every acronym correctly appears in parentheses after the full name.*

COMMENT #7:

The results should be further elaborated to show how they could be used for the real applications.

RESPONSE*: We expanded the discussion in Section 4.2, emphasizing limitations of our study and discussing issues related to the practical applications of our results, analyses and findings.*

REVIEWER #2

There were no comments from Reviewer #2.

REVIEWER #3

Wish a very good luck for all authors. The manuscript now is ready for publication after addressing all the comments.

RESPONSE*: We thank the reviewer #3 for kindful feedback and good wishes.*
